# Alarm calls of sagebrush converge when herbivory is high

**DOI:** 10.1098/rspb.2024.1513

**Published:** 2024-09-18

**Authors:** Richard Karban, Muhammad Usman Rasheed, Mikaela Huntzinger, Patrick Grof-Tisza, James Blande

**Affiliations:** ^1^ Department of Entomology & Nematology, University of California, Davis, CA 95616, USA; ^2^ Department of Environmental and Biological Sciences, University of Eastern Finland, Yliopistonranta 8, Kuopio 70211, Finland; ^3^ Department of Biology, Converse University, Spartansburg SC 29302, USA

**Keywords:** chemotypes, communication, eavesdropping, frequency-dependent, herbivory, volatile

## Abstract

Herbivory is a major threat to virtually all plants, so adaptations to avoid herbivory will generally be selected. One potential adaptation is the ability to ‘listen in’ on the volatile cues emitted by plants that are experiencing herbivory and to then respond by ramping up defences. The nature of these volatile cues is poorly understood. Sagebrush (*Artemisia tridentata*) plants that were exposed to cues of experimentally damaged neighbours experienced less herbivory; this induction was most effective if emitter and receiver plants had similar volatile emission profiles, termed chemotypes. Previously, we observed that sagebrush populations that were in locations with high herbivory exhibited little diversity of volatiles compared to populations with low herbivory. Several hypotheses could produce this correlation. High risk of herbivory could have selected for individuals that converged on a common ‘alarm cue’ that all individuals would respond to. In this case, individuals of locally rare chemotypes that were less able to eavesdrop would experience more damage than common chemotypes when herbivores were abundant. Alternatively, low chemotypic diversity could allow higher levels of damage to plants. In this case, rare chemotypes would experience less damage than common chemotypes. We examined the chemotypes of sagebrush individuals from multiple sites and found that rare chemotypes experienced more damage than common chemotypes when herbivores were abundant. This pattern was seen among sites and among years with different densities of herbivores. This result is consistent with the hypothesis that herbivory selects for individuals that are effective communicators and shapes the communication system.

## 1. Introduction

Herbivory can be an important ecological and evolutionary force for plants, and they have evolved diverse adaptations that act as defences. These defences are often costly, diverting resources from other fitness-related functions [1]. Plants reduce allocation and ecological costs by responding to reliable environmental cues to fine-tune their defensive phenotypes to current or likely future risks. Plants perceive and respond to a wide diversity of cues including tissue damage, changes in light levels, physical touch, chemicals associated with their herbivores and volatile chemicals emitted by damaged neighbouring plants [2,3].

There is now evidence that more than 30 plant species increase resistance to herbivores in response to the volatile emissions of damaged neighbours [4]. In some instances, these cues prime plants to respond more quickly or strongly to subsequent attacks, further reducing costs when defences against those attacks are not needed [5]. Specific cues also allow plants to target their responses to current threats, increasing their effectiveness. While we have considerable evidence that plants respond to volatile cues and that responses affect plant traits and herbivore performance, the cues involved are poorly understood [3,6].

Sagebrush (*Artemisia tridentata*) is the dominant plant in the Great Basin of North America, and it hosts a large diversity of herbivores over its extensive range [7,8]. Sagebrush is a slow-growing, long-lived perennial that is relatively intolerant of herbivory such that chewing herbivores can significantly reduce plant fitness [9,10]. Leaves of sagebrush that were experimentally damaged by chewing herbivores emitted a range of biologically active volatile compounds [11–13]. Most of these volatiles were produced constitutively and emitted in higher concentrations following damage to leaves. Exposure to these volatiles caused reduced damage in neighbouring undamaged tissues of the attacked plant, conspecifics and plants of other species [11,12,14,15].

Sagebrush branches that were incubated with volatiles from experimentally damaged neighbours increased expression of compounds that are assumed to provide defence [16] and experienced less chewing damage over the growing season [15]. However, branches of sagebrush are highly sectored [17] making induced resistance localized. Furthermore, the effects of volatile communication were only measurable over distances of 60 cm or less [15]. Plants that were experimentally inoculated with volatiles from clipped neighbours had higher survival and produced more new branches and inflorescences than controls when experiencing natural levels of herbivory [18].

The biologically active cues responsible for this communication are unknown and approximately 100 compounds have been identified from chromatograms of the headspace of experimentally damaged *Artemisia* spp. [19,20]. The volatile profiles of damaged plants vary among individuals at each site and across the landscape [4,13,20]. However, these profiles exhibit characteristic compounds that are repeated consistently among different individuals, which are termed chemotypes [21] (cited in [22]). Parent–offspring regressions have shown that chemotypes are highly heritable among sagebrush individuals (broad sense heritability for chemotype: *H*^2^ = 0.98 ± 0.36) [4]. Individuals of the same chemotype communicate to reduce herbivore damage approximately 25% more effectively compared to individuals of different chemotypes [4,16].

An examination of chemotypes from sagebrush populations on the eastern edge of the Sierra Nevada range in California and Nevada revealed variation in the diversity of chemotypes among populations [13]. We observed a negative relationship between chemotypic diversity at a site and the mean level of leaf damage by chewing herbivores at that site. Sites experiencing high levels of chewing damage had few chemotypes while those experiencing low damage had many chemotypes.

**Figure 1.**
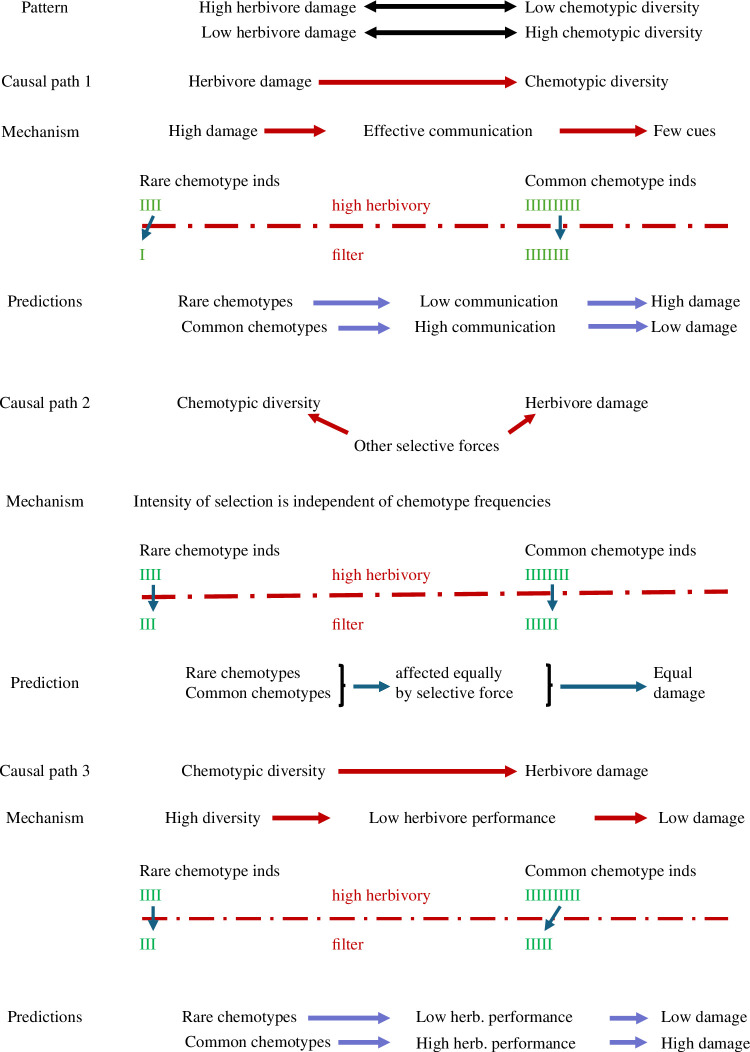
Three causal paths that could give rise to the correlational pattern that we observed between herbivore damage and chemotypic diversity. Each causal path would have a distinct mechanism and would lead to a distinct predicted outcome. Lines with arrows at both ends represent correlations; lines with arrows at one end represent directed causal paths; quinacridone red lines with one arrow represent selection; green I symbols represent the relative densities of plant individuals before and after a filter due to high levels of herbivory; the dashed red lines represent filters due to high herbivory. In all cases, high herbivory (red filter) leads to reduced plant abundances but at different relative rates. For causal path 1, high herbivory most negatively affects rare chemotypes; for causal path 2, herbivory affects common and rare chemotypes similarly; and for causal path 3, high herbivory most negatively affects common chemotypes.

This correlation between chemotypic diversity and herbivore load could result from several causal mechanisms (figure 1). Herbivory could potentially drive the diversity and nature of cues that plants use to recognize and respond to their environment (causal path 1 in figure 1). Communication may allow plants to escape damage more effectively and selection might favour plants that are able to interpret the cues of damage from most of their neighbours [23]. As a result of communication, individuals of the common chemotype may exhibit more appropriate defences and experience a benefit relative to those of rare chemotypes. This selection may be expected to be a more potent force where herbivory is consistently more intense. Where herbivory is weak, advantages of chemotypic diversity may have stronger selective influences. Under this scenario when herbivory is high, locally rare chemotypes would be expected to communicate less effectively and receive relatively more damage.

It is quite possible that other selective agents might influence both the diversity of chemotypes, and the level of damage caused by herbivores (causal path 2 in figure 1). For example, abiotic conditions might affect both the chemotypes that are present and the mean level of damage that plants experience. Under this scenario, communication between plants is not necessarily an important driver. There is no selection based on chemotypic frequency. High herbivory would be predicted to have similar effects on how much damage rare and common chemotypes experience.

Chemotypic diversity could potentially drive levels of herbivore damage (causal path 3 in figure 1). Many studies have found that higher diversity of host plant phenotypes reduces herbivore performance and levels of damage [24–28]. When herbivores are well matched to exploit the locally common chemotype, damage will be high. Under these favourable conditions for herbivores, locally rare chemotypes would be predicted to be at an advantage and to receive relatively less damage than common chemotypes. This advantage for rare chemotypes is independent of communication between plants.

The aim of this study was to evaluate these causal mechanisms by observing the levels of damage by chewing herbivores to individual sagebrush plants that exhibit relatively locally rare chemotypes compared to individuals with locally common chemotypes. These causal hypotheses make different predictions about the advantage or disadvantage experienced by rare chemotypes depending upon the overall level of herbivory.

## 2. Results

**Figure 2.**
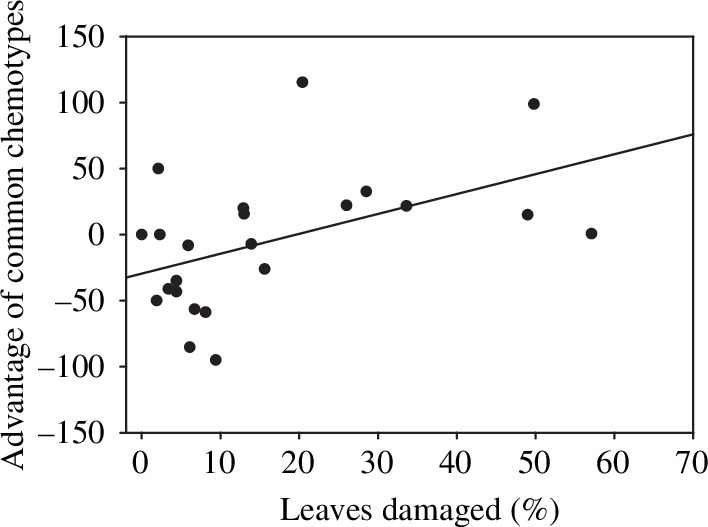
The relationship between mean level of chewing damage at a site (% of leaves with herbivory) and the relative advantage of the common chemotype ([mean damage to rare chemotypes – mean damage to common chemotypes] ÷ mean damage to all chemotypes) × 100). The line shows the best fit.

We identified 23 situations in which some individuals exhibited rare chemotypes (<15% of the local population) at sites where one or two chemotypes were common (>75% of the local population). Overall, there was no advantage in terms of relative amount of herbivore damage for individuals belonging to a common chemotype (mean advantage of having the common chemotype ± 1 s.e. = −2.6 ± 10.1 where advantage was measured as the percentage of reduction in leaves that were damaged). However, as levels of herbivore damage in a population increased, it became increasingly beneficial to belong to a common chemotype (figure 2; *R*^2^ = 0.24; *F*_1,21_ = 6.56, *p* = 0.02). In situations where conditions allowed herbivores to become abundant and cause more overall damage, rare chemotypes experienced relatively more damage than common chemotypes.

**Figure 3.**
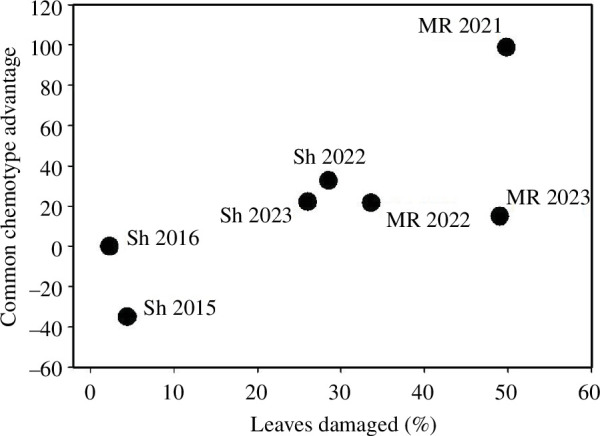
The relationship between mean level of chewing damage and the relative advantage of the common chemotype for plants at two sites (Sh = Sagehen and MR = Mt Rose) that were surveyed repeatedly over multiple years. Chewing damage at a site is percentage of leaves with herbivory and the relative advantage of the common chemotype is ([mean damage to rare chemotypes – mean damage to common chemotypes] ÷ mean damage to all chemotypes) × 100.

Two of the sites (Sagehen and Mt Rose) were surveyed over multiple years (4 years and 3 years, respectively), and these were considered as independent observations in our analysis. We believe that this is appropriate since each year represents a different situation for individual plants of rare and common chemotypes, depending upon the herbivores present, among other factors. However, if instead, the analysis is conducted with only a single mean value for level of herbivory for each site over those years (*x*-axis in figure 2) and for the advantage of the common chemotype (*y*-axis in figure 2), the result is qualitatively similar (*R*^2^ = 0.25; *F*_1,17_ = 5.74, *p* = 0.03). An analysis of those two sites considered in years with different conditions, and therefore different densities of herbivores, showed a similar trend; at each site (considered as random effects) as the mean level of herbivory increased, the relative advantage for plants of the locally common chemotypes also increased (figure 3; fixed effect of mean level of damage *F*_1,5_ = 16.57, *p* = 0.01).

## 3. Discussion

When herbivory was great, individuals of locally rare chemotypes experienced proportionally more damage than locally common chemotypes (figure 2). Communication that reduced damage was most effective between individuals of the same chemotypes [16,29]. Individuals of locally rare chemotypes were presumably less able to sense and respond to the cues of neighbours to defend against herbivores. We hypothesize that rare chemotypes that were less able to eavesdrop would have experienced increased mortality and reduced reproduction and would have been selectively eliminated when herbivory was high. Herbivory has been shown to be a potent selective force for this species [9,10]; we observed high rates of mortality at sites that were repeatedly defoliated by herbivores although we do not know if that mortality depended on chemotype. Our results contradict the hypothesis that rare chemotypes are advantageous when herbivory is intense. We found no evidence that exhibiting a locally common chemotype was advantageous when risk of herbivory was not great (figure 2). Under conditions of low herbivory, exhibiting a rare chemotype may be selectively neutral or even favoured if it allows individuals with rare chemotypes to experience fewer negative effects of competition with phenotypically similar neighbours.

One possible reason locally rare chemotypes may receive more herbivory compared to common chemotypes could involve the suitability of particular chemotypes to herbivores or other selective factors. The herbivores that happened to be abundant in one site might be particularly attracted to, or perform particularly well on, some chemotype relative to others. If this particularly susceptible chemotype happened to be rare, this scenario could produce the result that we observed. In this case, specific rare chemotypes could be at a disadvantage rather than rarity itself being disadvantageous. This explanation is unlikely to be sufficient to explain the result that we found (figure 2), as different rare chemotypes experienced high damage at different sites. The particular chemotype found at any site could have been the result of drift or selection due to other factors. Regardless of the reasons for their initial distributions, the disadvantage associated with rarity applied to many different chemotypes across the landscape. Conversely, the same chemotypes that were common at some sites were rare at others. For example, thujone and camphor were the common chemotypes at Sagehen and Brockway Summit, but they were the rare chemotypes at Mt Rose and Diamond Peak. Processes other than plant communication, such as attraction of predators or parasites of herbivores, could conceivably be causing the observed correlation as long as they were affected by both the frequency of chemotypes and the level of herbivory. However, predators that developed a preference or search image for the common chemotype would be expected to produce a result that favoured rare rather than common chemotypes.

Furthermore, at two of our sites (Sagehen and Mt Rose), plants were surveyed over multiple years. The advantage of having the common chemotype tended to increase as the overall level of damage increased (figure 3). This result suggests that particular common chemotypes do not perform uniformly better at particular sites but rather the advantage that common chemotypes accrue increases in situations when herbivore pressure is high.

Eavesdropping on volatiles emitted by damaged plants may have similarities to the alarm calls of animals. Vertebrates in high-risk situations often respond to the calls of conspecifics by freezing, seeking cover or mobbing the predator [30]. Many of these behaviours are innate and do not require learning. Animals are more likely to respond to the alarm calls of heterospecifics if the cues of the two species share common characteristics [31]. They are also more likely to respond if the information is reliable and relevant (e.g. if the predators are a risk to both species).

The influential Janzen–Connell hypothesis proposes that young plants that grow in high density close to their parents will experience higher rates of herbivory and mortality caused by host-specific herbivores compared to more widely dispersed individuals [32,33]. Density-dependent herbivory has been reported in recent reviews and meta-analyses supporting this hypothesis [34–36]. Janzen and Connell relied on specialization of herbivores to create this effect and subsequent studies have compared rates of herbivory for conspecific and heterospecific juveniles, as well as the phylogenetic distance between spatially close neighbours [34,36]. However, the survival of seeds and seedlings in the proximity of closely related parents has rarely been examined [35]. Furthermore, phenotypic and genotypic similarity within a species has not been considered in previous studies of this effect. Our result that locally uncommon chemotypes may experience less severe herbivory occurs at a finer, more intraspecific scale than the studies that support the Janzen–Connell hypothesis.

In conclusion, we previously found that sites with high levels of damage by herbivores exhibited low chemotypic diversity. In this study, chemotypes that were relatively rare at a site experienced more herbivory than those that were relatively common when herbivore pressure was high. This observation is consistent with the hypothesis that selection from herbivory favours individuals that can eavesdrop on neighbours and adjust levels of defences. Plots of goldenrod that were experimentally protected from herbivory exhibited greater chemotypic diversity than plots exposed to high herbivore pressure [23]. Our observational study suggests that similar processes occur at the landscape scale and that herbivory may contribute to shape plant communication systems.

## 4. Methods

### (a) Natural history

This study was conducted in three areas on the east side of the Sierra Nevada mountains in California and Nevada where we have worked and observed the relative levels of chewing damage to sagebrush plants during most years since 1995. Generalist grasshoppers were present at all sites and all years and generally chewed 3–10% of leaves at any site. Two specialist chrysomelid beetles, *Trirhabda pilosa* and *Monoxia grisea*, exhibited consistently higher densities at some sites that caused higher rates of chewing damage, completely defoliating and, in some instances, killing bushes. Once established, these high densities were evident for multiple years. Sites had characteristically low or high levels of damage that persisted over many years (personal observation). Other leaf-chewing herbivores that are known to outbreak, such as *Aroga websteri* (Lepidoptera: Gelechiidae) [37], have been seen occasionally during our study but never became common or caused much damage to their host plants.

We identified local sites with populations of sagebrush in the vicinity of University of California Natural Reserves at Sierra Nevada Aquatic Research Lab, Valentine Eastern Sierra Reserve, Sagehen Creek Field Station and Mt Rose in Humboldt-Toiyabe National Forest (electronic supplementary material, table S1 lists site locations). Of the local sites for which we had historical knowledge of relative rates of damage by chewing herbivores, 23 were dominated by sagebrush individuals with one or two common chemotypes that accounted for >75% of the local population and one or more rare chemotypes each of with accounted for <15% of the local population.

### (b) Chemotypes, damage and performance

Chemotypes were determined by collecting leaf samples which were shipped to the University of Eastern Finland for volatile collection and chemotypic determination following the protocol described by Grof-Tisza *et al.* [13]. Previous work demonstrated that shipping did not influence chemotypic determination [13]. Fresh leaves were chopped and placed into sealed 20 ml glass vials. Volatiles were collected using a network headspace sampler (Agilent G1888A, Agilent Technologies, Folsom, CA) connected to a network gas chromatograph (Agilent 6890N) and inert mass selective detector. Gas chromatography conditions were described by Grof-Tisza *et al*. [13] and methods used to assign chemotypes were described by Karban *et al*. [20]. We assigned chemotypes based on motifs of discriminating dominant compounds in the overall emission blend determined by gas chromatography–mass spectrometry. We found that the assignments of chemotypes using this method were the same as those determined by dynamic headspace sampling in the field and our assignments were confirmed by previous multivariate analyses [20,29].

We estimated the amount of chewing damage on 25–100 sagebrush plants (*Artemisia tridentata* ssp. *vaseyana*) at each site by examining 100 leaves on each plant and recording how many of those leaves had any biomass removed by herbivores. This estimate of the percentage of leaves damaged is positively correlated with leaf area removed [38].

We estimated chewing damage at 18 sites. At two of our field sites, we quantified rates of chewing damage for plants during multiple years. At Sagehen, the same marked plants were surveyed for damage in 2015, 2016, 2022 and 2023. At Mt Rose, plants were surveyed for damage in 2021, 2022 and 2023, and chemotype was determined although different plants were selected at this site in different years.

### (c) Statistical analysis

We estimated the advantage of belonging to the locally common chemotype(s) relative to the locally rare chemotype(s) at each site in terms of chewing damage that was accrued ([damage to rare chemotypes – mean damage to common chemotypes] ÷ mean damage to all chemotypes) × 100. This index was positive when rare chemotypes received more damage than common chemotypes and negative when common chemotypes received relatively more damage. The relationship between mean damage and the relative advantage for common chemotypes at each site was evaluated using a generalized linear model (GLM) (Fit Model in JMP Pro 17.1, SAS Institute, Cary, NC). The Sagehen and Mt Rose sites were sampled in multiple years. The relative advantage for common chemotypes at these two sites was analysed using a mixed-model GLM (JMP Pro 17.1) with site as a random effect and mean damage at that site in each year as a fixed effect.

## Data Availability

The data are presented in electronic supplementary material, table S2 [39].

## References

[1] Cipollini D, Walters D, Voelckel C. 2014 Costs of resistance in plants: from theory to evidence. Ann. Plant. Rev. 263–307. (10.1002/9781118829783)

[2] Karban R. 2015 Plant sensing and communication. Chicago, IL: University of Chicago Press.

[3] Brosset A, Blande JD. 2022 Volatile-mediated plant–plant interactions: volatile organic compounds as modulators of receiver plant defence, growth, and reproduction. J. Exp. Bot. **73**, 511–528. (10.1093/jxb/erab487) 34791168 PMC8757495

[4] Karban R, Yang LH, Edwards KF. 2014 Volatile communication between plants that affects herbivory: a meta-analysis. Ecol. Lett. **17**, 44–52. (10.1111/ele.12205) 24165497

[5] van Hulten M, Pelser M, van Loon LC, Pieterse CM, Ton J. 2006 Costs and benefits of priming for defense in Arabidopsis. Proc. Natl Acad. Sci. USA **103**, 5602–5607. (10.1073/pnas.0510213103) 16565218 PMC1459400

[6] Erb M. 2018 Volatiles as inducers and suppressors of plant defense and immunity-origins, specificity, perception and signaling. Curr. Opin. Plant Biol. **44**, 117–121. (10.1016/j.pbi.2018.03.008) 29674130

[7] Wiens JA, Cates RG, Rotenberry JT, Cobb N, Van Horne B, Redak RA. 1990 Arthropod dynamics on sagebrush (Artemisia tridentata): effects of plant chemistry and avian predation. Ecol. Monogr. **61**, 299–322. (10.2307/2937110)

[8] Sanford MF, Huntly NJ. 2010 Seasonal patterns of arthropod diversity and abundance on big sagebrush, Artemisia tridentata. West. North Am. Nat. **70**, 67–76. (10.3398/064.070.0108)

[9] Bilbrough CJ, Richards JH. 1993 Growth of sagebrush and bitterbrush following simulated winter browsing: mechanisms of tolerance. Ecology **74**, 481–492. (10.2307/1939309)

[10] Takahashi M, Huntly N. 2010 Herbivorous insects reduce growth and reproduction of big sagebrush (Artemisia tridentata). Arthropod Plant Interact. **4**, 257–266. (10.1007/s11829-010-9108-1)

[11] Farmer EE, Ryan CA. 1990 Interplant communication: airborne methyl jasmonate induces synthesis of proteinase inhibitors in plant leaves. Proc. Natl Acad. Sci. USA **87**, 7713–7716. (10.1073/pnas.87.19.7713) 11607107 PMC54818

[12] Kessler A, Halitschke R, Diezel C, Baldwin IT. 2006 Priming of plant defense responses in nature by airborne signaling between Artemisia tridentata and Nicotiana attenuata. Oecologia **148**, 280–292. (10.1007/s00442-006-0365-8) 16463175

[13] Grof-Tisza P, Karban R, Rasheed MU, Saunier A, Blande JD. 2021 Risk of herbivory negatively correlates with the diversity of volatile emissions involved in plant communication. Proc. R. Soc. B **288**, 20211790. (10.1098/rspb.2021.1790) PMC8548805 34702072

[14] Karban R, Baldwin IT, Baxter KJ, Laue G, Felton GW. 2000 Communication between plants: induced resistance in wild tobacco plants following clipping of neighboring sagebrush. Oecologia **125**, 66–71. (10.1007/PL00008892) 28308223

[15] Karban R, Shiojiri K, Huntzinger M, McCall AC. 2006 Damage-induced resistance in sagebrush: volatiles are key to intra- and interplant communication. Ecology **87**, 922–930. (10.1890/0012-9658(2006)87[922:drisva]2.0.co;2) 16676536

[16] Grof-Tisza P, Kruizenga N, Tervahauta AI, Blande JD. 2022 Volatile-mediated induced and passively acquired resistance in sagebrush (Artemisia tridentata). J. Chem. Ecol. **48**, 730–745. (10.1007/s10886-022-01378-y) 35984547 PMC9618528

[17] Cook CW, Stoddart LA. 1960 Physiological responses of big sagebrush to different types of herbage removal. J. Range Manag. **13**, 14–16. (10.2307/3894891)

[18] Karban R, Ishizaki S, Shiojiri K. 2012 Long‐term demographic consequences of eavesdropping for sagebrush. J. Ecol. **100**, 932–938. (10.1111/j.1365-2745.2012.01974.x)

[19] Lopes-Lutz D, Alviano DS, Alviano CS, Kolodziejczyk PP. 2008 Screening of chemical composition, antimicrobial and antioxidant activities of Artemisia essential oils. Phytochemistry **69**, 1732–1738. (10.1016/j.phytochem.2008.02.014) 18417176

[20] Karban R, Grof-Tisza P, Blande JD. 2016 Chemotypic variation in volatiles and herbivory for sagebrush. J. Chem. Ecol. **42**, 829–840. (10.1007/s10886-016-0741-8) 27525992

[21] Santesson R. 1968 Lavar: some aspects of lichen taxonomy. Sv. Nat. Vetensk. **21**, 176–184.

[22] Keefover‐Ring K, Thompson JD, Linhart YB. 2009 Beyond six scents: defining a seventh Thymus vulgaris chemotype new to southern france by ethanol extraction. Flav. Fragr. J. **24**, 117–122. (10.1002/ffj.1921)

[23] Kalske A, Shiojiri K, Uesugi A, Sakata Y, Morrell K, Kessler A. 2019 Insect herbivory selects for volatile-mediated plant-plant communication. Curr. Biol. **29**, 3128–3133.(10.1016/j.cub.2019.08.011) 31522939

[24] Elton CS. 1958 The ecology of invasions. London, UK: Chapman and Hall. (10.1007/978-1-4899-7214-9)

[25] Johnson MT, Lajeunesse MJ, Agrawal AA. 2006 Additive and interactive effects of plant genotypic diversity on arthropod communities and plant fitness. Ecol. Lett. **9**, 24–34. (10.1111/j.1461-0248.2005.00833.x) 16958865

[26] Hughes AR, Inouye BD, Johnson MTJ, Underwood N, Vellend M. 2008 Ecological consequences of genetic diversity. Ecol. Lett. **11**, 609–623. (10.1111/j.1461-0248.2008.01179.x) 18400018

[27] Wetzel WC, Kharouba HM, Robinson M, Holyoak M, Karban R. 2016 Variability in plant nutrients reduces insect herbivore performance. Nature **539**, 425–427. (10.1038/nature20140) 27749815

[28] Pearse IS, Paul R, Ode PJ. 2018 Variation in plant defense suppresses herbivore performance. Curr. Biol. **28**, 1981–1986.(10.1016/j.cub.2018.04.070) 29887306

[29] Karban R, Wetzel WC, Shiojiri K, Ishizaki S, Ramirez SR, Blande JD. 2014 Deciphering the language of plant communication: volatile chemotypes of sagebrush. New Phytol. **204**, 380–385. (10.1111/nph.12887) 24920243

[30] Magrath RD, Haff TM, Fallow PM, Radford AN. 2015 Eavesdropping on heterospecific alarm calls: from mechanisms to consequences. Biol. Rev. **90**, 560–586. (10.1111/brv.12122) 24917385

[31] Fallow PM, Gardner JL, Magrath RD. 2011 Sound familiar? Acoustic similarity provokes responses to unfamiliar heterospecific alarm calls. Behav. Ecol. **22**, 401–410. (10.1093/beheco/arq221)

[32] Janzen DH. 1970 Herbivores and the number of tree species in tropical forests. Am. Nat. **104**, 501–528. (10.1086/282687)

[33] Connell JH. 1971 On the role of natural enemies in preventing competitive exclusion in some marine animals and in rain forest trees. Dynam. Popul. **1970**, 298–312.

[34] Comita LS, Queenborough SA, Murphy SJ, Eck JL, Xu K, Krishnadas M, Beckman N, Zhu Y, Gómez-Aparicio L. 2014 Testing predictions of the Janzen–Connell hypothesis: a meta-analysis of experimental evidence for distance- and density-dependent seed and seedling survival. J. Ecol. **102**, 845–856. (10.1111/1365-2745.12232) 25253908 PMC4140603

[35] Basset Y, Miller SE, Gripenberg S, Ctvrtecka R, Dahl C, Leather SR, Didham RK. 2019 An entomocentric view of the Janzen–Connell hypothesis. Insect Conserv. Divers. **12**, 1–8. (10.1111/icad.12337)

[36] Terborgh J. 2020 At 50, Janzen–Connell has come of age. Bioscience **70**, 1082–1092. (10.1093/biosci/biaa110)

[37] Gates RC. 1965 Sagebrush defoliator outbreak in northern california. Berkeley, CA: US Forest Service.

[38] Karban R, Yang LH. 2020 Feeding and damage-induced volatile cues make beetles disperse and produce a more even distribution of damage for sagebrush. J. Anim. Ecol. **89**, 2056–2062. (10.1111/1365-2656.13270) 32472554

[39] Karban R, Rasheed MU, Huntzinger M, Grof-Tisza P, Blande JD. 2024 Data from: Alarm calls of sagebrush converge when herbivory is high. Figshare. (10.6084/m9.figshare.c.7430647) PMC11407867 39288807

